# Supplementary material for: Investigating the influence of physiologically relevant hydrostatic pressure on CHO cell batch culture
Source: Sci Rep. 2021 Jan 8;11:162. doi: 10.1038/s41598-020-80576-8 (PMC7794228; doi:10.1038/s41598-020-80576-8)
Supplement: Supplementary file 1 — Supplementary Information 1. [file 41598_2020_80576_MOESM1_ESM.zip › S1/IgG/Feb14 0 30 60 90/0mmHg Jan30.pdf]

Data File C:\Users\P...hemStation\1\Data\TK_CHO_IgG 2019-02-14 10-52-34\008-5-ML 0 Jan30.D
Sample Name: ML 0 Jan30

===

Acq. Operator : SYSTEM   Seq. Line : 8
Sample Operator : SYSTEM
Acq. Instrument : HPLC-0039   Location : 5
Injection Date : 2/14/2019 11:24:26 AM   Inj : 1
                                          Inj Volume : 100.000 µl
Method : C:\Users\Public\Documents\ChemStation\1\Data\TK_CHO_IgG 2019-02-14 10-52-34\CHO_IgG_LC.M (Sequence Method)
Last changed : 2/8/2019 3:17:23 PM by SYSTEM

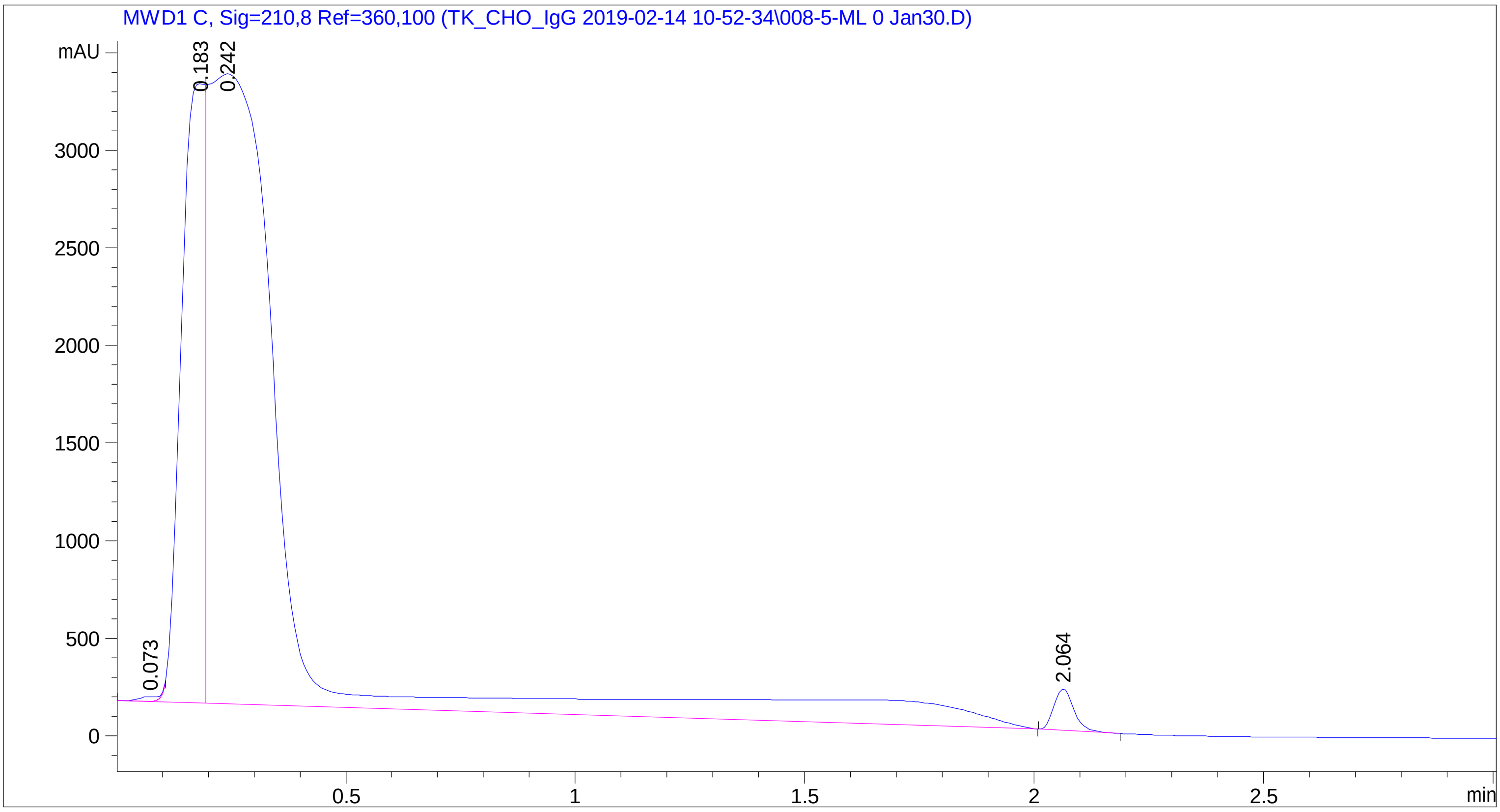

===

Area Percent Report

===

Sorted By : Signal
Multiplier : 1.0000
Dilution : 1.0000
Use Multiplier & Dilution Factor with ISTDs

Signal 1: MWD1 C, Sig=210,8 Ref=360,100

Peak #	RetTime [min]	Type	Width [min]	Area [mAU*s]	Height [mAU]	Area %
1	0.073	BV E	0.0407	69.06195	24.65605	0.1402
2	0.183	VV R	0.0554	1.12585e4	3172.15479	22.8525
3	0.242	VB	0.1728	3.72982e4	3228.16406	75.7080
4	2.064	BBA	0.0472	640.14459	212.42146	1.2994

Totals : 4.92659e4 6637.39636

===

*** End of Report ***
